# Bucking the trend: understanding lipopolysaccharide structure and outer membrane dynamics in cold-adapted *Pseudomonas* isolated from Enigma Lake, Antarctica†

**DOI:** 10.1039/d4sc05116e

**Published:** 2024-09-18

**Authors:** Marcello Mercogliano, Stefania De Chiara, Antonio De Nicola, Jacopo Cardellini, Costanza Montis, Mikhail M. Yakimov, Violetta La Cono, Francesca Crisafi, Alba Silipo, Debora Berti, Giuseppe Milano, Antonio Molinaro, Flaviana Di Lorenzo

**Affiliations:** a Department of Chemical Science, University of Naples Federico II Via Cinthia, 4 Naples 80126 Italy flaviana.dilorenzov@unina.it; b Cineca Casalecchio di Reno (BO) 40033 Italy adenicola.chem@gmail.com; c Research Center for Organic Electronics (ROEL), Yamagata University 4-3-16 Jonan Yonezawa Yamagata 992-8510 Japan; d Consorzio Interuniversitario Per Lo Sviluppo Dei Sistemi A Grande Interfase Via Della Lastruccia 3 Firenze 50019 Italy costanza.montis@unifi.it; e Dipartimento Di Chimica “Ugo Schiff”, Università Degli Studi di Firenze Via Della Lastruccia 3 Firenze 50019 Italy; f Institute of Polar Sciences, National Research Council of Italy (ISP-CNR) Via S. Raineri 4 Messina 98122 Italy; g CEINGE-Biotecnologie Avanzate Franco Salvatore Via Gaetano Salvatore 486 Napoli 80145 Italy; h Department of Chemical, Material and Production Engineering, University of Naples Federico II Piazzale V. Tecchio, 80 Naples 80125 Italy; i Department of Chemistry, School of Science, Osaka University 1-1 Osaka University Machikaneyama Toyonaka Osaka 560-0043 Japan

## Abstract

Cold environments are predominant over the Earth and are inhabited by bacteria able to cope with a series of simultaneous environmental pressures. Gram-negative species of the *Pseudomonas* genus are the predominant ones isolated from cold habitats, making them an excellent model for studying the mechanisms of bacterial adaptation to the most extreme habitats on our planet. Here we focused on the lipopolysaccharide (LPS) structure and the outer membrane dynamics of *Pseudomonas* sp. EW#7, a strain isolated from Enigma Lake in Antarctica where, among other extreme characteristics, water temperature can reach 0.4 °C. We show that near-zero growth temperature mostly affects the LPS lipid A component. An uncommon tendency of decreasing lipid A secondary hydroxylation while increasing its phosphorylation degree was observed. This resulted in a faster lateral diffusion of lipid chains in the membrane and therefore in an enhancement of its fluctuations that guarantee membrane integrity and flexibility.

## Abstract 2

*Pseudomonas* from Enigma Lake (Antarctica) has a peculiar lipopolysaccharide with an unusual tendency of decreasing lipid A secondary hydroxylation. This results in increased membrane fluidity that ensures bacterial survival at near-zero temperature.

## Graphical abstract

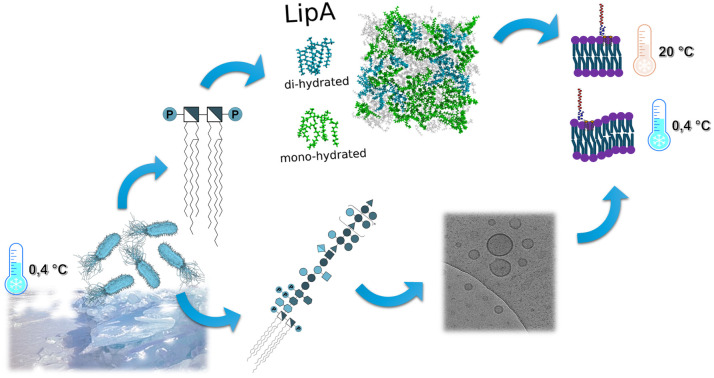

## Introduction

*Pseudomonas*, which currently is the genus of Gram-negative bacteria with the largest number of species, is an excellent example of widespread microorganisms able to adapt to various growth conditions and complex environments, comprising terrestrial, freshwater, marine ecosystems as well as the human body.^1^ This remarkable versatility makes *Pseudomonas* a perfect research model to explore the influence of environmental factors on chemical and physical properties of the bacterial outer membrane. Since growth temperature significantly impacts both on structural and biological properties, seminal studies focused on the analysis of *Pseudomonas* strains grown at different temperatures revealing an optimal growth temperature in the range 25–30 °C.^2–5^ Nevertheless, low-temperature habitats are ideal sites for the colonization of several *Pseudomonas* species, which make them among the predominant ones isolated from cold environments.^6^ Considerable interest has been developed and progress has been made to understand how *Pseudomonas* spp. acclimatize to low temperature, thus disclosing a number of survival strategies based on bacteria physiological changes.^7^ These include the production and release of anti-freeze proteins and cryoprotectants, alteration of metabolic processes and acquisition of energy *via* alternate routes. Certainly, one key tactic for survival at low temperatures is to ensure membrane integrity and functionality by controlling the fluidity of the membrane itself.^7^ As a matter of fact, *Pseudomonas* typically reshapes membrane fluidity by altering the lipid polar head, charge, location and size, length and distribution of fatty acid chains.^8^ For example, it has been extensively reviewed that an increased amount of low-melting-point fatty acids, which comprises monounsaturated, polyunsaturated and branched-chain fatty acids, results in an improved membrane fluidity compared to membranes constituted by their saturated straight analogs.^9–11^ Of note, transcriptome analyses have demonstrated that exposure to cold temperatures induces up-regulation of specific genes involved in outer membrane biogenesis, such as fatty acid and lipopolysaccharide (LPS) biosynthesis, whereas the expression of genes encoding other outer membrane structures, like flagella, is mostly suppressed.^12^

As the main constituent of the *Pseudomonas* outer membrane, LPS heavily contributes to the structural integrity and fluidity of the cell envelope, thus providing resilience against external stressors.^13^ The dynamic properties of LPSs are strictly related to their chemical structure, which includes a glycolipid anchor to the membrane, the lipid A, a core oligosaccharide (core OS), and a polysaccharide chain, termed O-antigen.^13^ An LPS made up of these three components is defined smooth-type LPS (or S-LPS), whereas when the polysaccharide moiety is absent the terminology employed is rough-type LPS (or R-LPS).^14^ Contextually, bacteria typically modify the inner core OS-lipid A area to enhance the integrity of the outer membrane. This is achieved through changes in the composition of acyl chains, phosphate groups, and other functional groups.^15^ Likewise, modulation of the strength of membrane bilayers through O-antigen expression was also investigated by molecular coarse-grained Molecular Dynamics (MD) simulations, which evidenced how the lamellar packing of O-antigen chains affects lipid mobility within the membrane and confers an increased capacity to withstand significant surface tension.^16,17^

In this work we focused on a specific cold adapted strain *Pseudomonas* sp. EW#7, which was isolated from water column of the permanently ice-covered Enigma Lake (Antarctica).^18,19^ Of note, the main water masses of the Antarctica Enigma Lake present unique extreme conditions characterized by strong alkalinity, oxygen oversaturation, and a temperature that does not exceed 0.6 °C.^20^ The uniqueness of this microbial isolation boosted our interest in investigating the structure and molecular characteristics of the outer membrane and its LPS constituent from such an intriguing polyextremophile. Specifically, we have isolated LPS from *Pseudomonas* sp. EW#7 grown at two different temperatures, *i.e.* 0.4 °C (*in situ* temperature of the collected water of Enigma Lake) and 20 °C (referred to hereafter as LPS_0.4C_ and LPS_20C_). We have defined their chemical structures, the morphology of their assemblies in water, the viscoelastic properties of LPS membranes, and the effects of their lipid A in membrane rigidity. An uncommon tendency of decreasing lipid A hydroxylation while increasing its phosphorylation was observed for the bacterium grown at 0.4 °C, which resulted in a faster lateral diffusion of lipid chains and therefore in an enhanced membrane fluctuation responsible for membrane flexibility and integrity preservation.

## Results and discussion

### LPS isolation and structural characterization

**Fig. 1:**
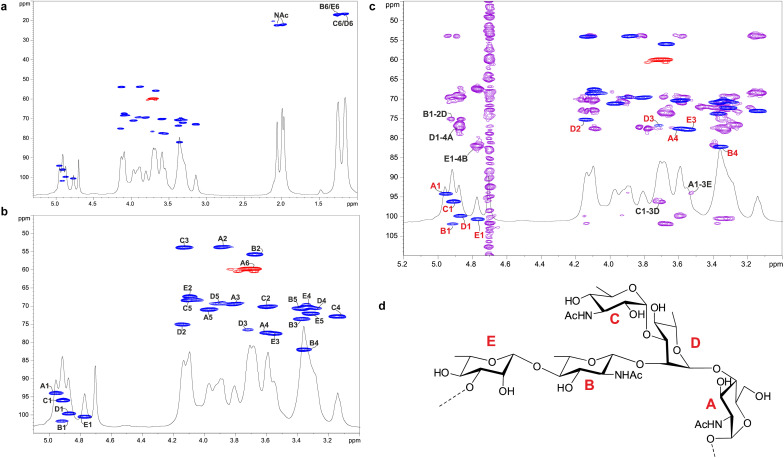
Structural characterization of *Pseudomonas* sp. EW#7 O-antigen. (a) ^1^H DOSY and ^1^H, ^13^C HSQC NMR spectra of the O-antigen fraction obtained upon mild acid hydrolysis followed by size-exclusion chromatography of LPS from *Pseudomonas* sp. EW#7 grown at 0.4 °C. (b) Zoom of the carbinolic region. Key heteronuclear one-bond correlations are indicated. (c) Section of the superimposed ^1^H DOSY, ^1^H, ^13^C HMBC (purple) and ^1^H, ^13^C HSQC (blue and red) NMR spectra of the O-antigen from *Pseudomonas* sp. EW#7 grown at 0.4 °C. The key inter-residue long–range correlations involving sugar moieties are indicated. (d) Structure of the O-antigen repeating unit defined for both *Pseudomonas*. Numbering of sugar residues is as reported in Table S1.

Enigma Lake is the largest and deepest perennially frozen lake occurring in the Northern Foothills, Northern Victoria Land in Antarctica.^18,19,21^ This peculiar lake, whose name is due to the presence of an unusual debris-laden area in its middle, hosts a large variety of microorganisms, like *Pseudomonas* species, able to withstand harsh conditions. As a matter of fact, *Pseudomonas* species are widely recognized as one of the most metabolically diversified bacterial colonizers of cold habitats.^6,22^ Therefore, we have isolated *Pseudomonas* sp. EW#7 grown at 0.4 °C and 20 °C and characterized the structure and morphology of its membrane. LPS_0.4C_ and LPS_20C_ were isolated from lyophilized bacterial pellets through the hot phenol-water extraction method^23^ and then extensively purified by means of enzymatic digestion and ultracentrifugation. Purified LPS fractions were both inspected *via* SDS-PAGE followed by silver nitrate staining, which revealed that both *Pseudomonas* produce a smooth-type LPS. Chemical analyses^24–26^ highlighted an identical sugar and lipid composition. In detail, monosaccharide analyses revealed the presence of l-rhamnose (l-Rha), 2,6-dideoxy-2-amino-l-glucose (l-quinovosamine, l-QuiN) and 3,6-dideoxy-3-amino-d-glucose (d-Qui3N), 2-amino-2-deoxy-d-glucose (d-GlcN), and in minor amount d-glucose (d-Glc), 2-amino-2-deoxy-d-galactose (d-GalN), l-*glycero*-d-*manno*-heptose (l,d-Hep), and 3-deoxy-d-*manno*-oct-2-ulopyranosonicacid (Kdo). Linkage analysis revealed the occurrence mainly of 2,3-disubstituted l-Rha and 3-substituted l-Rha, 4-substituted-d-GlcN, 4-substituted-l-QuiN, terminal d-Qui3N. As stated above, fatty acids composition, which was obtained by GC-MS analysis of the related methyl ester derivatives, showed the same composition for both LPSs. However, a higher abundance of 2-hydroxylated acyl moieties was noticed for LPS_20C_ compared to LPS_0.4C_. In detail, in both LPSs it was highlighted the presence of *R*-3-hydroxydodecanoic acid [12:0(3-OH)] and *S*-2-hydroxydodecanoic acid [12:0(2-OH)], *R*-3-hydroxydecanoic [10:0(3-OH)] and dodecanoic acid (12:0), which was in agreement with lipid A fatty acid composition found for other *Pseudomonas* strains.^27,28^ To define the structure of the carbohydrate and lipid portion, a mild acid hydrolysis was performed on an aliquot of both purified LPS_0.4C_ and LPS_20C_ to selectively cleave the linkage between the non-reducing GlcN of the lipid A and the Kdo. The lipid A fractions were then inspected by MALDI-TOF MS and MS/MS while the carbohydrate portions were further purified by size-exclusion chromatography and then analyzed *via* 1D and 2D NMR. The NMR investigation revealed that bacteria grown at different temperatures express the same O-antigen repeating unit that was fully characterized. In detail, the ^1^H, ^1^H DOSY and ^1^H, ^13^C HSQC NMR spectra of the carbohydrate portion of both LPS_0.4C_ and LPS_20C_ (Fig. 1a, b and S1†) highlighted the presence of five anomeric signals, diagnostic for five spin systems (A–E, Table S1†). The structural assignment of each spin system was performed by tracing the spin connectivity visible in the COSY and TOCSY spectra, while the assignment of carbon atoms was deduced by analyzing the HSQC spectrum. The anomeric configuration of each monosaccharide unit was defined tracing the *intra*-residue NOE correlations observable in the NOESY spectrum.^29^ In detail, residues A, B and C were identified as *gluco*-configured sugar units as inferred by the efficient propagation of magnetization in the TOCSY spectrum and by their ^13^C chemical shift values, with C-4 of A and B (77.2 and 82.0 ppm, respectively, Table S1†) shifted at low field by glycosylation. An α-anomeric configuration was deduced for residues A and C, while B was a β-configured sugar unit as proven by the evident *intra*-residue NOE correlation between its H-1, H-3 and H-5. The observation that from the anomeric proton of B and C it was possible to identify connections up to the methyl groups (H-6) at 1.27 ppm (B) and 1.14 ppm (C), along with the correlation of the H-2 of B and H-3 of C with two nitrogen-bearing carbon atoms (55.8 and 53.8 ppm), led to their identification as QuiN and Qui3N, respectively. Likewise, residue A was a terminal GlcN assigned on the basis of its H-2 proton that correlated with a nitrogen-bearing carbon atom at 53.8 ppm. All the three amino sugar residues (A-C) also bear *N*-acetyl groups (Table S1†). Spin systems D and E were assigned to rhamnoses, as indicated in the TOCSY spectrum by the scalar correlations from H-2 to H-6 methyl protons (1.16 and 1.26 ppm). D and E were α- and β-configured respectively at the anomeric center on the basis of the agreement between their C-5 values (69.3 and 72.1 ppm) and the value reported for the related *O*-methyl rhamnoside (69.4 ppm for α anomer, 73.6 for β anomer).^29^ Downfield displacement of carbon signals revealed substitution at O-2 and O-3 of D and at O-3 of E. The primary sequence of the O-antigen repeating unit was then established by tracing both *inter*-residue NOE contacts and the long–range correlations visible in the ^1^H,^13^C HMBC (Fig. 1c and S2†). Briefly, β-l-QuiNAc residue B was found to be substituted at its position O-4 by β -l-Rha E, in turn substituted at its position O-3 by α-d-GlcNAc A, as proven by the related NOE contact between H-1 E and H-4 B and H-1 A and H-3 E (Fig. S2†). Residue A was in turn substituted at position O-4 by α-l-Rha D that carried the terminal α-d-Qui3NAc C at position O-3 and β-l-QuiNAc B at position O-2, as demonstrated by the detection of the related HMBC correlations (Fig. 1c). Therefore, the O-antigen repeating unit was defined as the branched pentasaccharide reported in Fig. 1d. These results led to the conclusion that the growth temperature did not affect the carbohydrate portion of LPS, as both *Pseudomonas* sp. EW#7 produced the same O-antigen repeating unit.

**Fig. 2:**
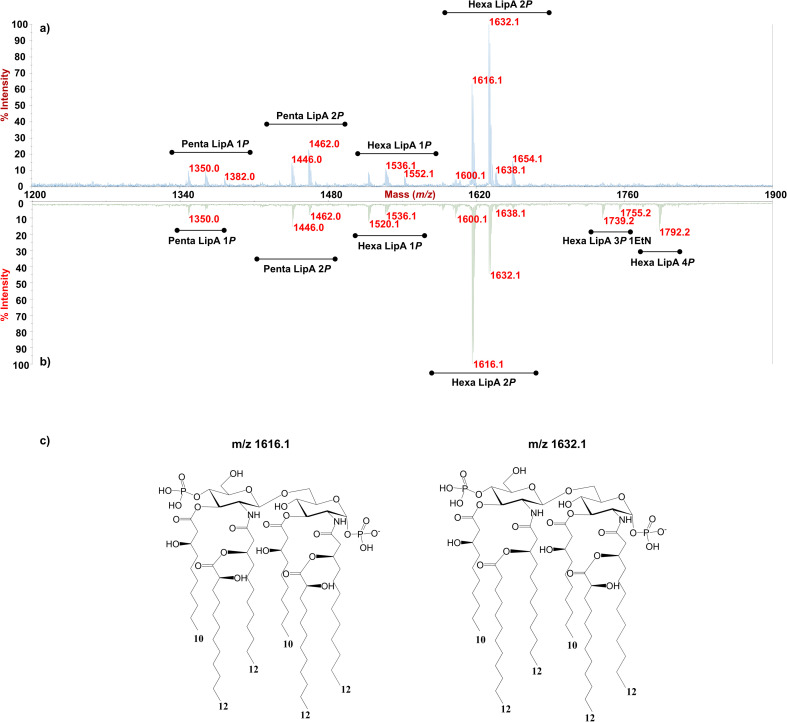
MALDI-TOF MS spectra of the lipid A from *Pseudomonas* sp. EW#7 grown at 20 °C (a) and grown at 0.4 °C (b). Penta- and Hexa-acylated lipid A species were labelled as Penta- and HexaLip A indicating the degree of acylation. “1*P*”, “2*P*” and “4*P*” are indicative of the mono-, bis-and tetra-phosphorylated lipid A species, respectively. *P*EtN indicates the 2-amino ethyl phosphate group. (c) Structure of the bis-phosphorylated hexa-acylated lipid A structures detected *m/z* 1616.1 (left) and *m/z* 1632.1 (right).

As for the lipid A moiety, negative-ion MALDI-TOF MS spectra were recorded for both LPSs highlighting key structural differences. As shown in Fig. 2, both *Pseudomonas* produced a mixture of mono and bis-phosphorylated penta- and hexa-acylated lipid A species. For both lipid A variants, MALDI-TOF MS spectra displayed three distinct peaks with differences of 16 amu, due to the presence or absence of a hydroxyl group in the secondary C12 acyl substituents, although different relative abundances were observable between the two spectra. In fact, the main lipid A species identified for the bacterium grown at 20 °C (Fig. 2a) was detected at *m/z* 1632.1 and assigned to bis-phosphorylated hexa-acyl lipid A bearing four 12:0(OH) and two 10:0(OH), whereas the main species for the *Pseudomonas* sp. EW#7 grown at 0.4 °C (Fig. 2b) was detected at *m/z* 1616.2 and matched with bis-phosphorylated hexa-acyl lipid A bearing three 12:0(OH), two 10:0(OH), and one 12:0. Minor peaks at −16 amu (*m/z* 1600.1), detected in both spectra, indicated the absence of a hydroxyl group on the acyl chains. Therefore, a reduction in the lipid A hydroxylation state was observed when growth temperature was dramatically decreased to 0.4 °C. Mono- and bis-phosphorylated penta-acylated forms were also identified and were assigned to species devoid of a C10:0(OH) moiety. Besides the hydroxylation pattern, a slight decrease in the relative intensity of peaks corresponding to penta-acylated lipid A species was also noticed when *Pseudomonas* sp. EW#7 was grown at low temperature. Notably, only when *Pseudomonas* sp. EW#7 was grown at 0.4 °C it was possible to identify an additional cluster of peaks that matched with bis-phosphorylated hexa-acylated species further decorated by a 2-aminoethyl phosphate group (at *m/z* 1755.2) and by two additional phosphate groups (*m/z* 1792.2). Negative-ion MALDI-TOF MS/MS analysis of the main species provided specific fragmentation patterns that guided the structural characterization of lipid A structures (Fig S3 and Table S2†), which agreed with previous structures from *Pseudomonas* sp., and confirmed a higher degree of secondary hydroxylation of fatty acid chains for LPS_20C_ compared to LPS_0.4C_. According to chemical analyses, it is plausible to conclude that LPS_20C_ and LPS_0.4C_ are built with the same core OS structure, although it remains to be defined. Therefore, the growth temperature seems to exclusively affect the lipid A portion in *Pseudomonas* sp. EW#7, producing at 0.4 °C lipid A species with a lower degree of secondary 2-hydroxylation than at 20 °C, but with a higher degree of phosphorylation. These results were extremely interesting as previous studies have highlighted an increment of hydroxylated fatty acids in LPS from another cold-adapted *Pseudomonas*, *i.e. P. syringae*, at low temperature compared to 22 °C temperature-grown cells, with differences affecting only primary acyl chains and not secondary 12:0 (2-OH).^30^ In addition, *Pseudomonas* sp. EW#7 did not produce lipid A species carrying unsaturated or branched acyl chains, which are well known strategies employed by bacteria to provide an adequate membrane fluidity in harsh and highly variable habitats.^9,31–35^ As it has been reviewed by Hassan *et al.*,^9^*Pseudomonas* is the most dominant genus isolated from cold environments, followed by *Arthrobacter*, *Brevundimonas*, and *Massilia*, whose cell membrane fatty acids, in 24 out of 42 strains, were mainly straight-chain monounsaturated, whereas 18 species were found to have branched acyl chains, both saturated and monounsaturated.^9^ Among these, *P. mandelii* and *P. veronii* displayed the highest percentage of unsaturated acyl chains and *P. frederiksbergensis* the highest percentage of branched fatty acids.^9^ In addition, the alteration of LPS phosphorylation in response to high and low temperatures is acknowledged as aiding modulation of membrane function and consequently help in cold-temperature adaptation. On the contrary, previous studies on *P. syringae* showed that LPS was phosphorylated more at 22 °C and less at 4 °C, although most of this increment in phosphorylation was associated with the core OS region.^36^

### Cryo-EM and SAXS characterization of *Pseudomonas* sp. EW#7 LPS_0.4C_ and LPS_20C_

To glean molecular information about LPS_0.4C_ and LPS_20C_, we first investigated structure and morphology of both LPS assemblies in water *via* Cryo-EM images and Small-Angle X-ray Scattering (SAXS) experiments, which are complementary techniques (in the direct and reciprocal space, respectively) enabling structural characterization at the sub-micron scale.

**Fig. 3:**
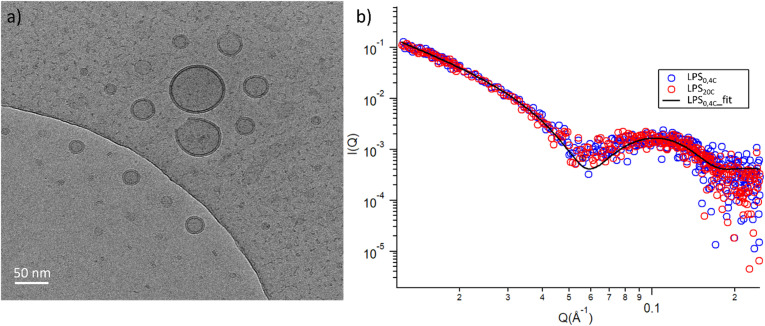
(a) Cryo-EM micrographs of 1 mg mL^−1^ LPS water solution; (b) log–log SAXS profiles of 1 mg mL^−1^ water dispersion of LPS_0.4C_ (red circles) and LPS_20C_ (blue circles).

Both LPS_0.4C_ and LPS_20C_ were dispersed in water at a concentration of 1 mg mL^−1^ (see experimental section). As shown, in Fig. 3a, LPS_0.4C_ and LPS_20C_ dispersion assembled into vesicle-like structures, featuring a well-defined bilayer that separates an inner aqueous pool from the external medium. It is worth noting that a minority of spherical and rod-like structures without the bilayer were also visible (Fig. S4†). However, although being very informative on the shape and size of aggregates, Cryo-EM may be not fully representative of the whole sample, since only a few assemblies are imaged. To fill this gap and to identify possible structural differences between the LPS_0.4C_ and LPS_20C_ at the ensemble-average level, SAXS was used to assess the overall shape of LPS assemblies. The scattering intensity *I*(*Q*) as a function of the scattering vector (*Q*) is reported in Fig. 3b. SAXS profiles originating from the scattering of LPS_0.4C_ and LPS_20C_ (red circles and blue circles, respectively) were almost superimposable. This experimental result clearly indicated that the differences in the LPS molecular structures (*i.e.* in the lipid A moiety) did not significantly affect the overall structures of the aggregates. To get additional molecular insights, the SAXS curves were analyzed through a bilayered vesicle model with the SASfit software package.^37^ According to this model, the inner and outer layers of hydrophilic heads (*t*_h_) are considered as two concentric shells with identical scattering length density (SLD), separated by an additional shell of hydrophobic tails (*t*_t_) with a lower SLD with respect to the solvent. The SLD values inserted in the model fitting were consistent with recent literature reports.^38^ Fitting parameters (Table S3†) and further details about SAXS measurements are reported in the ESI.† This analysis confirmed the locally bilayered shape of LPS_0.4C_ and LPS_20C_ assemblies. The total bilayer thickness (*t*_t_ + 2 *t*_h_) for LPS_0.4C_ and LPS_20C_ was in agreement with literature values evaluated for several LPS membranes.^39–41^ Overall, SAXS and Cryo-EM results highlighted that the molecular differences in the LPS molecular structures do not affect the overall morphology of their assemblies in water and slightly affect the bilayer thickness.

To shed light on the viscoelastic properties of LPS membranes, we collected lateral–pressure isotherms of the LPS films at the air water-interface, as LPS monolayers have proven to be accurate, albeit simple model interfaces to simulate the behavior of the outer membranes of Gram-negative bacteria.^42,43^

**Fig. 4:**
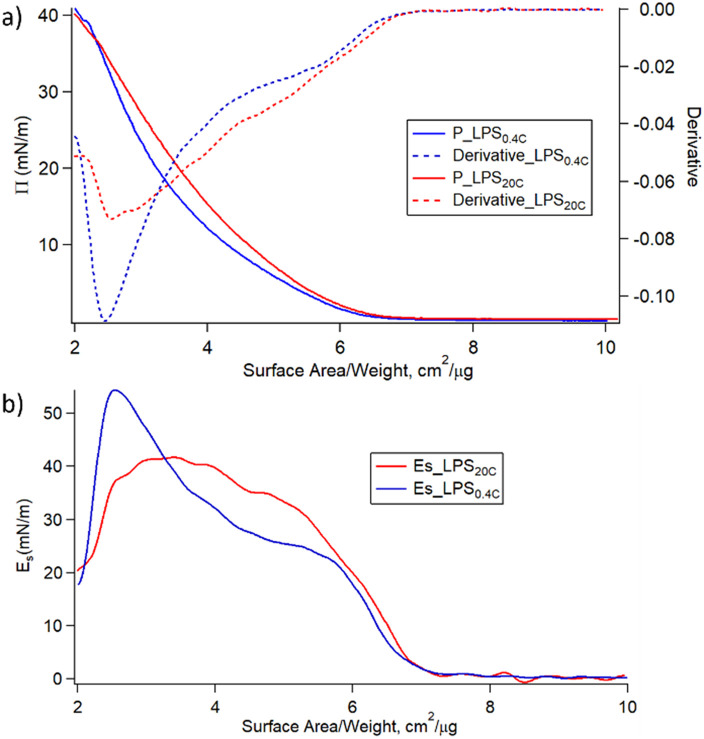
(a) Surface pressure-area isotherms (solid lines) and curves derivatives (dashed lines) of LPS_20C_ (red) and LPS_0.4C_ (blue); (b)compression modulus (*E*_s_) as a function of area per unit weight (A) for LPS_20C_ (red) and LPS_0.4C_ (blue).

Fig. 4a shows the variation of the surface pressure (π) as a function of the air–water interfacial area for LPS_0.4C_ and LPS_20C_. Further compression leads to the increase of π until the film collapses occurring at 35–40 mN m^−1^ for both LPS molecules. Such low lateral pressure values of the collapse of the monolayers highlights the high water solubility of LPS monomers. When the pressure increases, the LPS monomers desorb from the air–water interface, eventually forming the spontaneous assemblies observed in SAXS and Cryo-EM measurements. Additionally, the monolayer compressibility modulus (*E*_s_) was evaluated as a function of the area per unit weight and is reported in Fig. 4b. Initially, *E*_s_ increases similarly for the two LPSs. Then, for higher values of lateral pressure (5 mN m^−1^ < π < 25 mN m^−1^) the compressibility, and in turn, the rigidity of the LPS_20C_ monolayer, overcomes the one of LPS_0.4C_. However, when approaching pressure values relevant for membrane conditions (25 mN m^−1^ < π < 30 mN m^−1^), the compressibility moduli of the monolayers assume similar values for the two LPS. Reasonably, this evidence suggests that, in these experimental conditions, the compositional differences between the two LPSs slightly affect the rigidity of the monolayer. The same trends of isotherms and, in turn, similar behavior of compressive moduli were obtained during the compression of heterogeneous monolayers composed of LPS, DOPE, and DSPE, selected as more realistic compositional mimics of Gram-negative outer membranes (Fig. S5†). As a more general consideration, the compressibility values obtained at membrane pressures relevant for membrane conditions (*E*_s_ = 40 mN m^−1^) correspond to the formation of liquid-expanded (LE) films,^43^ suggesting the formation of soft LPSs monolayers. Accordingly, as shown in Table 1, the compressibility of LPS_0.4C_ and LPS_20C_ are significantly lower compared to compressibility moduli obtained for *E. coli* LPS monolayers and literature values obtained for different LPSs (Fig. S5†).^44,45^

**Table 1:** *E*_s_ moduli of LPS_0.4C_ and LPS_20C_ at relevant pressure membrane conditions compared to *E*_s_ calculated for LPS from *E. coli* (see also Fig. S5) and literature values of wild-Type LPS Ca^2+^-free, wild-Type LPS Ca^2+^-loaded, and LPS from *Pseudomonas aeruginosa*

LPS	*E*_s_ (mN m^−1^)
LPS_0.4C_	40
LPS_20C_	40
LPS *E. coli*	65
Wild-type LPS Ca^2+^-free^32^	80
Wild-type LPS Ca^2+^-loaded^32^	99
LPS *Pseudomonas aeruginosa*^31^	225

### Molecular dynamics simulations

**Fig. 5:**
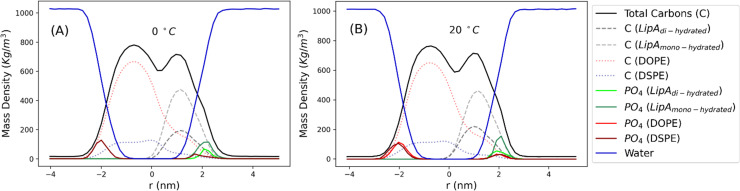
Mass density profiles calculated for the mix systems (see Table 2 for composition details) simulated at 0 and 20 °C. Profiles have been calculated averaging over the last 250 ns of production runs.

**Fig. 6:**
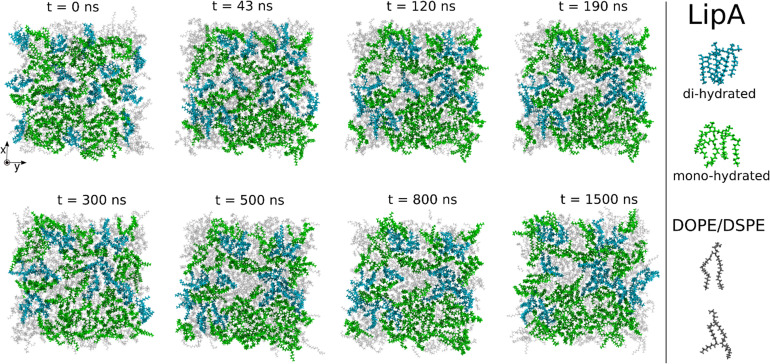
Sequence of top-view snapshots of the mixed system (see Table 2) simulated at 20 °C. All lipids are shown only in the upper layer of the membrane. LipA_mono-hydrated_ molecules are depicted in green, LipA_di-hydrated_ molecules are shown in blue, and DSPE and DOPE phospholipids are both represented in light gray. A similar behavior is observed in the simulations of the mixed system at 0 °C.

**Fig. 7:**
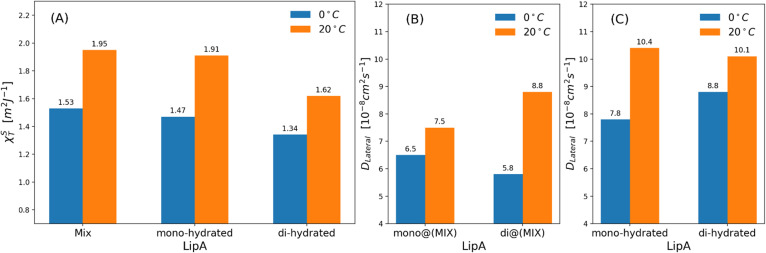
(a) Isothermal area compressibility calculated for lipid membranes as function of lipid A composition and temperatures. The calculations have been made considering the last 500 ns of production runs. Lateral diffusion *D*_Lateral_ calculated at two different temperatures and for: (b) mixed systems (see Table 2), (c) systems in which Lipids A are exclusively mono-hydrated or di-hydrated. The time behavior of area per lipid is reported, for all systems, in Fig. S7.

Intrigued by the diverse degree of secondary 2-hydroxylation observed for LPS_0.4C_ and LPS_20C_, we next used MD computational technique to evaluate whether the structural differences in the glycolipid anchor of the two LPSs impact on membrane properties. For simplicity, we chose to investigate the most representative bis-phosphorylated hexa-acyl lipid A structures, as experimentally identified, adopting a full atom representation of the molecules. Computational details and atomistic model description are reported in the ESI section.† To understand the effects of hydroxylation on membrane properties, in terms of the lipid A chemical distinctions, three different lipid bilayer systems were constructed. The lower layers of the three systems were composed exclusively by DOPE and DSPE phospholipids, while for the upper layers we set: one having exclusively a single secondary 2-hydroxylated acyl substitution (referred to as LipA_mono-hydrated_), one presenting two 2-hydroxylated acyl substitutions (LipA_di-hydrated_) and one comprising a mixture of both types (LipA_mix_). The choice to have different lipid compositions of the simulated systems has been taken to closely match the estimated distribution of mono and bis-phosphorylated hexa-acylated lipid A produced by *Pseudomonas* in low temperature conditions. In Table 2, the composition of simulated system is reported, while in Fig. S6† a pictorial view of the difference in the lipid A composition at low and higher temperature is shown. From MD simulations, we extracted structural information about distribution of molecules in the cross-section direction of the bacteria membrane plane, enabling us to compute the thickness of the hydrophobic core of and compared it with experimental estimations made by SAXS measurements. Specifically, simulations yielded a hydrophobic tail thickness of approximately 2.2 nm at both temperatures, which is close to the experimentally measured value of 2.8 nm. The overall membrane thickness, calculated as the peak-to-peak distance of PO_4_ groups, was found to be 4.1 nm for the system simulated at 20 °C, and 4.2 nm for the system at 0 °C. The results from MD simulations match the experimental indication about the little structural differences between the two bilayers growth at low and higher temperature. Calculated mass density profiles are reported in Fig. 5. Visual inspection of the MD runs revealed a common behavior at the simulated temperatures regarding the interactions between mono- and di-hydrated LipA in the mixed systems. Starting from a random initial distribution of LipA_di-hydrated_ in the upper layer of the bacterial membrane, we observed the formation of small aggregates of LipA_di-hydrated_ as the simulation progressed. Notably, once these small aggregates (comprising 2 or 3 LipA molecules) are formed, they remained stable longer and diffused as larger single units. The picture of this behavior can be gained looking at the time sequence of LipA molecules reported in the snapshots of Fig. 6. The snapshots sequence of the upper-layer shows the formation of stable LipA_di-hydrated_ clusters (top-view of LipAs). Above 300 ns, clusters of LipA_di-hydrated_ (depicted in light blue) are formed and remain stable for the whole simulated time. The presence of these stable clusters can potentially affect the behavior of the lateral diffusion of both mono- and di-hydrated LipA, as well as membrane area fluctuations, thereby altering membrane fluidity. To explore this phenomenon, we have exploited the concept that rigidity or fluidity of a membrane is linked to the amount of energy required to deform it. This can be expressed as the work needed to compress or expand a membrane (referred to as the Helmholtz free energy change) and from this relationship we could calculate the isothermal area compressibility (see the Area Compressibility Calculation section in the ESI†). Through MD simulations, it is possible to directly compute the isothermal area compressibility, providing insights into membrane rigidity. Through this approach, we have reported in Fig. 7a the membrane area fluctuations calculated from MD runs. As observed in Fig. 7a, the isothermal area compressibility remained almost unchanged for systems with a higher percentage of LipA_mono-hydrated_ (Table 2), but slightly higher than systems comprised solely of LipA_di-hydrated_. This observation suggested an increased flexibility for membranes containing a higher proportion of LipA_mono-hydrated_, in alignment with experimental observations and monolayer compressibility modulus measurements.

**Table 2:** Composition of simulated systems. Upper and Lower indicate the membrane layer. *Lipid A populates solely the upper leaflet of the bilayer. CaCl_2_ is added to make the systems electrically neutral

System	Lipid A*		DOPE	DSPE	Water	Box [nm]	Temp. [°C]
	Mono-hydrated	Di-hydrated					
LipA0.4mix	52	23	21 (upper)	4 (upper)	16 107	9.64804	0
LipA0.4mono-hydrated	75	—	84 (lower)	16 (lower)		9.64804	
LipA0.4di-hydrated	—	75				8.79987	
LipA20mix	45	30	21 (upper)	4 (upper)	16 107	9.77029	20
LipA20mono-hydrated	75	—	84 (lower)	16 (lower)		9.77029	
LipA20di-hydrated	—	75				8.45202	

Interestingly, the decrease of the isothermal area compressibility (a reduction of about 17–26%) due to the reduction of temperature from 20 °C to 0 °C was modest. For the sake of comparison, the isothermal area compressibility of a DPPC membrane decreases from to 6.9–7.5 m^2^ J^−1^ (at 25 °C) to 1.1 m^2^ J^−1^ (at 8 °C), indicating, in this case, a strong temperature-dependence and a drastic change of membrane fluidity.^46,47^ The results here reported clearly show how membrane fluidity of LipA_mix_ is preserved, thus allowing for membrane integrity at low temperature by means of modulation of lipid A chemistry. Finally, we calculated lateral self-diffusion coefficient *D*_L_ from MD simulations because *D*_L_ is correlated to the translational membrane fluidity (further details in the ESI†). In Fig. 7b and c, *D*_L_ is computed and averaged over all LipA molecules belonging to the upper leaflet of the membrane. *D*_L_ coefficients were calculated based on 2 μs-long trajectories. The bar plot in Fig. 7b, in the case of the mixtures, revealed an inversion in lateral diffusion for LipA_mono-hydrated_, which exhibited faster diffusion (mono@(MIX) in Fig. 7b*D*_L_ = 6.5 × 10^−8^ cm^2^ s^−1^) than LipA_di-hydrated_ di@(MIX) in Fig. 7b.

*D*_L_ = 5.8 × 10^−8^ cm^2^ s^−1^) at low temperature (0 °C), but a slightly slower diffusion (mono@(MIX) in Fig. 7b*D*_L_ = 7.5 × 10^−8^ cm^2^ s^−1^), compared to LipA_di-hydrated_ (di@(MIX) in Fig. 7b*D*_L_ = 8.8 × 10^−8^ cm^2^ s^−1^) at higher temperature (20 °C). Differently, in systems where the Lipid A composition is exclusively LipA_mono-hydrated_ or LipA_di-hydrated_, the lateral diffusion LipA_mono-hydrated_ at 0 °C (*D*_L_ = 7.8 × 10^−8^ cm^2^ s^−1^) is lower than that of LipA_di-hydrated_ (*D*_L_ = 8.8 × 10^−8^ cm^2^ s^−1^), while at 20 °C, both Lipid As show similar *D*_L_ values (see Fig. 7c). These results indicate that the significant decrease in lateral diffusion at 0 °C and 20 °C for LipA_di-hydrated_ in the mixed systems is somehow mitigated by the *D*_L_ of LipA_mono-hydrated_, which is less affected by temperature changes. Additionally, given that the composition of the mixed system at lower temperatures is richer in LipA_mono-hydrated_ compared to the composition at 20 °C, we can speculate that this contributes to preserve the membrane area fluctuations, thereby maintaining membrane fluidity.

## Conclusions

Enigma Lake in Antarctica is home to a unique microbial community able to face the strongly alkalinity, oxygen oversaturation and near-zero temperatures of this habitat.^20^ Among these microorganisms, we have isolated *Pseudomonas* sp. EW#7. Structural and morphological characterization of membrane of bacteria inhabiting Enigma Lake is crucial for understanding the essential components that contribute to their innate adaptive capacity to cope with cold and its associated stresses. In this perspective, we have focused on the main constituent of *Pseudomonas* sp. EW#7 outer membrane, the LPS, and have defined its chemical structure, the morphology of LPS assemblies in water, the viscoelastic properties of LPS membranes, as well as the impact of its lipid A component in membrane rigidity. To do this, we have isolated the LPS from *Pseudomonas* sp. EW#7 grown at 0.4 °C (LPS_0.4C_) and compared to the LPS from the same bacterium grown at 20 °C (LPS_20C_). We have disclosed that the growth temperature did not affect the carbohydrate portion of LPS, as both *Pseudomonas* sp. EW#7 produced the same O-antigen repeating unit, which was identified as a novel pentasaccharide made up of Rha, QuiNAc, Qui3NAc and GlcNAc (Fig. 1d). Indeed, also our SAXS and Cryo-EM results highlighted a similar morphology of LPS_0.4C_ and LPS_20C_ assemblies in water, likewise the mechanical properties of the LPS monolayer at the air–water interface resulted similar. On the contrary, structural differences were identified in the lipid A portion, surprisingly showing that *Pseudomonas* sp. EW#7 tends to decrease the degree of lipid A secondary hydroxylation to grow at 0.4 °C. Our molecular dynamics studies have evidenced that, at low temperature, lipid As carrying only one secondary hydroxylation (LipA_mono-hydrated_) exhibit faster lateral diffusion than lipid As with two secondary 12:0 (2-OH) (LipA_di-hydrated_), thus facilitating membrane fluctuations and consequently preserving membrane flexibility and integrity. Of note, and still against the trend, lipid A from *Pseudomonas* sp. EW#7 grown at 0.4 °C also displayed a more heterogenous phosphorylation pattern, with minor species carrying four phosphate groups or three phosphates and one EtN group, which were not produced by the bacterium when grown at 20 °C. Therefore, although only present as minor species, our data demonstrates that *Pseudomonas* sp. EW#7 may adopt lipid A hyperphosphorylation as a strategy to adapt to the extremely cold environmental temperature.

Overall, the combination of experimental and computational approaches enabled us to investigate the systems at multiple levels of resolution, providing a more comprehensive understanding of the structural and dynamic properties of the LPS membranes. However, more in-depth studies will be conducted to understand the full significance of *Pseudomonas* sp. EW#7 lipid A phosphorylation in the physiology of its outer membrane. In addition, whether these structural differences are species-dependent or are due to the growth temperature of 0.4 °C, *i.e.* lower than the typical 4 °C used in other studies, remains to be defined.

## Data availability

The data supporting this article have been included as part of the ESI.†

## Author contributions

A. M. conceived the study. A. M., A. S. and F. D. L. designed the research. M. M. and S. D. C. extracted, purified the LPS and analyzed the data. F. D. L. run MS and NMR studies. A. D. N. and G. M. run computational studies. J. C., C. M. and D. B. performed SAXS and Cryo-EM analyses. M. M. Y., V. L. C and F. C. provided bacterial cells. All the authors contributed to analyzing the data and writing the manuscript and have given approval to the final version of the manuscript.

## Conflicts of interest

There are no conflicts to declare.

## Supplementary Material

SC-OLF-D4SC05116E-s001
