# Supplementary material for: Bucking the trend: understanding lipopolysaccharide structure and outer membrane dynamics in cold-adapted Pseudomonas isolated from Enigma Lake, Antarctica
Source: Chem Sci. 2024 Sep 18;15(43):17852–61. doi: 10.1039/d4sc05116e (PMC11421494; doi:10.1039/d4sc05116e)
Supplement: SC-015-D4SC05116E-s001 [file SC-015-D4SC05116E-s001.pdf]

Supplementary Information (SI) for Chemical Science.
This journal is © The Royal Society of Chemistry 2024

Supplementary Information

Bucking the trend: understanding lipopolysaccharide structure and outer membrane dynamics in cold-adapted *Pseudomonas* isolated from Enigma Lake, Antarctica

Experimental and Computation methods

Supplementary Tables

Table S1. ^{1}H and ^{13}C of the chemical shift values of the O-antigen fraction

Table S2. The main ion peaks observed in the MALDI-TOF MS spectrum and assignment

Table S3. Fitting parameters from SAXS data fitted with bilayered vesicle model from Sasfit.

Supplementary Figures

Figure S1. ^{1}H NMR spectra of the O-antigen fractions

Figure S2. ^{1}H, NOESY and COSY spectra of the O-antigen

Figure S3. Negative-ion MS/MS spectra of precursor ions at *m/z* 1616.1 and *m/z* 1632.1

Figure S4. Cryo-EM micrographs of 1 mg/mL LPS water solution

Figure S5. Surface pressure-area isotherms and curves derivatives

Figure S6. Schematic representation of the upper- and lower-layers compositions for the mix systems

Figure S7. Time behavior of area/lipid A_0

Supplementary References

Experimental and Computational Methods

Isolation and cultivation of Pseudomonas sp. EW#7

Sampling was carried out during the XXXV Italian Expedition to Antarctica (November 2019-January 2020) in the perennially ice-covered and highly stratified Lake *Enigma*, located in the ice-free area of Northern Foothills (Northern Victoria Land, Antarctica). A water sample was taken from drilling point #4 (-74°42.956', 164°1.037') at a depth of 11m, which corresponds to a sharp thermocline layer. The thickness of the ice shield over the lake at this sampling point was 9.6 meters. The main physicochemical parameters of the collected sample, recorded *in situ*, were as follows: temperature - 0.4°C; dissolved oxygen - 48 mg L^{-1}; pH 10.05; total salinity - 570 mg L^{-1}. For cultivation, 100 µL of collected lake water was diluted 10 times with the same water, prefiltered sequentially through Millex®-GS 0.22 µm and Whatman® Anotop 0.1 µm syringe filters (both from Merck). 10 µL of resulting dilution was directly plate onto solidified by addition of Bacto agar (1.5 % w/v, Merck) minimal microbial growth medium M9 (Sigma-Aldrich), supplemented with yeast extract, peptone, casamino acids and sucrose (300 mg L^{-1} each). Before sterilization, the pH of the culture medium was adjusted to 8.7 using 10 M of NaOH. Cultivation was performed at 4 °C and pure cultures obtained on plates after two weeks of incubation were transfer to fresh plates. Colony transfer of strain EW#7 was repeated at least twice before the cultures were considered pure. Isolates were checked microscopically and by 16S rRNA sequencing. Phylogenetic analysis of the obtained 16S rDNA was found to be 99.68% identical to both *Pseudomonas gessardii* MT626825 and *Pseudomonas fluorescens* LC507955. *Pseudomonas* sp. EW#7 strain was than grown routinely in liquid Rich Enigma Medium (REM) (1g L^{-1} of yeast extract, 1g L^{-1} of LB and 1g L^{-1} of Na-pyruvate) buffered with 5 g L-1 of TAPSO (pH 8.7). Then, to obtain sufficient amount of biomass for LPS isolation, growth was carried out at both 0.4 °C (*in situ* temperature of collected Enigma Lake water) and 20 °C in 6-liter flasks filled with 3,000 mL of REM with stirring. Bacterial batch cultures were monitored using a photometer (Eppendorf BioPhotometer). When the OD600 reached value of ≈ 1, corresponding to the early stationary phase, the biomass of both cultures was collected by centrifugation (30 min, 10,000 x *g*) and then lyophilized.

Isolation and purification of Pseudomonas sp. EW#7 LPS

The LPS of both bacteria grown at different temperatures was isolated from dried cells through the hot phenol/water extraction method.[1,2] Both extracts were dialyzed (cutoff 12–14 kDa) against distilled water, then were subjected to an enzymatic treatment with DNAse (Roth), RNAse (Roth) (8 h, 37 °C) and proteases (Roth) (16 h, 56 °C) followed by extensive dialysis (cutoff 12–14 kDa) against distilled water. Digested LPS fractions were then ultracentrifuged (100,000 × *g*, 4 °C, 16 h) and further purified on a gel-filtration chromatography Sephacryl High Resolution S-300

(GE-Healthcare, Little Chalfont, UK) column eluted with 50 mM ammonium bicarbonate. The nature of the purified extracts was inspected by SDS-PAGE after gel staining with silver nitrate (Sigma Aldrich) according to the standard protocols. Fractions containing LPS were pooled and lyophilized.

Compositional analysis of Pseudomonas sp. EW#7 LPS

Monosaccharide residues were detected as acetylated *O*-methylglycoside derivatives (AMG) obtained after methanolysis (1.25 M hydrogen chloride in methanol, 85 °C, 16 h) followed by acetylation with acetic anhydride in pyridine (85 °C, 30 min). The so-obtained AMG were analyzed by Gas chromatography/Mass Spectrometry (GC-MS) and compared with opportunely prepared standards.[3] The absolute configuration of each sugar unit was defined through the analysis of the acetylated *O*-octylglycoside derivatives (AOG), as previously reported.[4] To determine the sugar linkage pattern of both LPSs, a derivatization to partially methylated and acetylated alditols (PMAA) of the monosaccharides followed by GC-MS analysis was performed. Briefly, an aliquot of dried samples was suspended in DMSO in presence of a tip of spatula of NaOH alternating stirring and sonication for 2 h at room temperature, and then it was treated with iodomethane. Then, a hydrolysis with trifluoroacetic acid (2 M, 100 °C, 4 h), carbonyl-reduction by employment of $NaBD_4$, and acetylation with acetic anhydride in pyridine, were executed.
Fatty acid content was established by treating an aliquot of both LPSs with HCl (4 M,100 °C, 4 h), followed by a treatment with NaOH (5 M, 100 °C, 30 min). After adjustment of the pH (~ 3), fatty acids were extracted in chloroform, methylated with diazomethane, and analyzed by means of GC-MS.[5] The ester-bound fatty acids were released after treatment with 0.5 M aqueous NaOH/CH3OH (1:1, v/v, 85 °C, 2 h); the products were then acidified, extracted in chloroform, methylated with diazomethane, and inspected by GC-MS. Finally, an aliquot of LPS was also treated with hydrogen chloride in methanol (1.25 M, 85 °C, 16 h) followed by extraction in hexane which contained the fatty acids as methyl esters derivatives (FAME); these were then analyzed via GC-MS. The absolute configuration of hydroxylated fatty acids was established as previously described through their conversion into the methoxy acid L-phenylethylamides, and then analyzed by GC-MS. All the above derivatives were analyzed on Agilent Technologies Gas Chromatograph 7820A equipped with a mass selective detector 5977B and an HP-5 capillary column (Agilent, Milan, Italy 30 m × 0.25 mm i.d., flow rate 1 mL/min, He as carrier gas). The temperature program used to inspect AMG and OGA was: 140 °C for 3 min, then 140 → 240 °C at 3 °C/min. The temperature program for PMAA was: 90 °C for 1 min, then 90 → 140 °C at 25 °C/min, then 140 → 200 °C at 5 °C/min, then 200 → 280 °C at 10 °C/min, and finally 280 °C for 10 min. To analyze FAME temperature program used was: 150 °C for 5 min, 150 to 280 °C at 3 °C/min, and 280 °C for 5 min.

Isolation of the carbohydrate and lipid A fractions

To establish the complete structure of the saccharide and lipid A portions, an aliquot of each sample was dissolved in acetate buffer (pH 4.4, 2 h, 100 °C) under constant magnetic stirring. A mixture of CH_3OH and $CHCl_3$ was added to the acid hydrolysis product to reach a $CH_3OH/CHCl_3$/hydrolysate 2:2:1.8 (v/v/v) ratio. The mixture was then shaken and centrifuged (4 °C, 8,800 × *g*, 20 min). Then, the chloroform phases, containing the lipid A fractions, were collected and washed with the water phase of a freshly prepared Bligh/Dyer mixture ($CHCl_3/CH_3OH/H_2O$, 2:2:1.8).[5] The organic phases were pooled, dried, and inspected by MALDI-TOF MS to define the chemical structure of the lipid A. The water phases, containing the carbohydrate fractions, were collected separately and lyophilized. These were then purified by size-exclusion chromatography on a Biogel P4 (Biorad) eluted with distilled water. Both saccharide fractions were analyzed by means of 1D and 2D NMR spectroscopy.

NMR spectroscopy

1D and 2D ^{1}H NMR spectra were recorded in D2O at 298 K at pD = 7 with a Bruker 600 AVANCE NEO instrument equipped with a cryoprobe. The spectra were calibrated with internal acetone (δH = 2.225 ppm; δC = 31.45 ppm). Total correlation spectroscopy (TOCSY) experiments were recorded with spinlock times of 100 ms by using data sets (t1 × t2) of 4096 × 256 points. Rotating frame Overhauser enhancement spectroscopy (ROESY) and Nuclear Overhauser enhancement spectroscopy (NOESY) experiments were performed by using data sets (t1 × t2) of 4096 × 256 points and by using mixing times between 100 and 300 ms, acquiring 16 scans. Double-quantum-filtered phase sensitive correlation spectroscopy (DQF-COSY) experiments were carried out by using data sets of 4096 × 512 points. The data matrix in all the homonuclear experiments was zero-filled in both dimensions to give a matrix of 4K × 2K points and was resolution-enhanced in both dimensions by a cosine-bell function before Fourier transformation. Coupling constants were determined by 2D phase-sensitive DQF-COSY. Heteronuclear single quantum coherence (^{1}H, ^{13}C HSQC) and heteronuclear multiple bond correlation (^{1}H, ^{13}C HMBC) experiments were executed in ^{1}H-detection mode by single-quantum coherence with proton decoupling in the ^{13}C domain using data sets of 2048 × 256 points. ^{1}H, ^{13}C HSQC was performed using sensitivity improvement and in the phase-sensitive mode using Echo/Antiecho gradient selection, with multiplicity editing during selection step. ^{1}H, ^{13}C HMBC was optimized on long range coupling constants, with low-pass *J*-filter to suppress one-bond correlations, using gradient pulses for selection. Moreover, a 60 ms delay was used for the evolution of long-range correlations. ^{1}H, ^{13}C HMBC was optimized for a 6 Hz coupling constant. The data matrix in all the heteronuclear experiments was extended to 2048 × 1024 points by using forward linear prediction extrapolation.

MALDI-TOF MS and MS/MS analysis of lipid A fractions

MALDI-TOF MS spectra were recorded on an ABSCIEX TOF/TOF™ 5800 Applied Biosystems mass spectrometer equipped with an Nd:YAG laser (λ = 349 nm), with a 3 ns pulse width and a repetition rate of up to 1000 Hz and also equipped with delayed extraction technology. For isolated lipid A fractions, the matrix solution was prepared by dissolving trihydroxyacetophenone (THAP) in CH_3OH/0.1% TFA/CH_3CN (7:2:1, v/v/v) at a concentration of 75 mg/mL, while the samples were dissolved in $CHCl_3$/CH_3OH (1:1, v/v). However, since lipid As might interact differently with diverse organic solvents, other two matrices were used, i.e. DHB and 5-chloro-2-mercaptobenzothiazole, which at the same laser strength, provided MS spectra comparable to those recorded by using THAP. In addition, different ratios between the samples and matrix were tested, all by using fast-drying conditions, with 1:1 providing best signal-to-noise ratio. Lipid A from commercially available *E. coli* 0127:B8 LPS (Sigma-Aldrich), obtained by home-made mild acid hydrolysis, was used as a standard. For MS experiments, acquired in negative polarity, reflectron mode, each spectrum was a result of the accumulation of 3000 laser shots, whereas 5000–7000 shots were summed for the MS/MS spectra. Spots were randomly but evenly sampled for the range *m/z* 1200-1900. All experiments were executed in technical triplicate and repeated on six aliquots of lipid A (three for each strain) obtained as a result of three independent mild acid hydrolysis reactions conducted on both isolated LPSs.[6,7]

Small Aangle X-Ray Scattering

The SAXS curves were analyzed through a *bilayered vesicle model* with the SASfit software package. The whole set of employed fit parameters is reported in the Table S3. The obtained thicknesses of hydrophobic tail layer (t_t) and hydrophilic head layers (t_h) for $\mathbf{LPS_{0.4C}}$ were t_t=2.8 nm and t_h=2.3 nm, respectively, while for $\mathbf{LPS_{20C}}$ they were t_t=2.8 nm and t_h=2.2 nm, corresponding to a total bilayer thickness (t_t+2 t_h) for $\mathbf{LPS_{0.4C}}$ and $\mathbf{LPS_{20C}}$ of 7.4 nm and 7.2 nm, respectively.

The monolayer compressibility modulus (E_s), that quantitatively describes the monolayer rigidity,[8] can be extrapolated from the isotherms:

$$E_s = -A\left(\frac{d\pi}{dA}\right) \qquad (1)$$

Where A is the area per unit weight.

Cryo-EM

On glow-discharged Quantifoil Cu 300 R2/2 grids were applied 3 µL of 1 mg/mL of $\mathbf{LPS_{0.4C}}$ and $\mathbf{LPS_{20C}}$ water dispersions. Briefly, 1 mg/mL of an organic solution of each LPS was spread on the water subphase, and the organic phase was left evaporating

at 20 °C to obtain a homogenous LPS film at the air-water interface. Then, LPS films were compressed at a controlled rate while recording the lateral film pressure. Once the packing area reaches approximately 7 $cm^2/\mu g$, the monolayers pressure increases due to intermolecular LPS-LPS interaction at the air-water interphase. The specimens were plunge frozen in liquid ethane using an FEI Vitrobot Mark IV (Thermo Fisher Scientific) instrument. Excess liquid was removed by blotting for 1 s (blot force of 1) using filter paper under 100% humidity and 10 °C. Cryo-EM data were collected at the Florence Center for Electron Nanoscopy (FloCEN), University of Florence, on a Glacios (Thermo Fisher Scientific) instrument at 200 kV equipped with a Falcon III detector operated in the counting mode. Images were acquired using EPU software with a physical pixel size of 2.5 Å and a total electron dose of ~ 50 e–/Å2 per micrograph.

SAXS Measurements

Small Angle X-Ray Scattering profiles were measured on $\mathbf{LPS_{0.4C}}$ and $\mathbf{LPS_{20C}}$ water dispersions using a Xeuss 3.0HR (Xenocs) instrument equipped with a Genix3D (Cu) X-Ray source and a Dectris 1 M Eiger detector. Samples were contained in glass capillary tubes of thickness 1.5 mm. Data from each sample were acquired at Sample-Detector (S-D) distance of 1800 mm for 600 s. Intensities were normalized with respect to transmission and sample thickness. After data reduction, the contribution of the sample holder and solvent (water) was subtracted from the sample intensity. The data were analyzed with the Sasfit software. The scattering intensity I_{BLV} from SAXS was fitted with the Bilayered Vesicle model:

$$I_{BLV}(Q) = -n_h + K(Q, R_c + t_t + t_h, n_h - n_t) + K(Q, R_c + 2t_t + t_h, n_t - n_{sol}{}^2 \quad (2)$$

with:

$$K(Q,R,\Delta\eta) = \frac{4}{3}\pi R^3 \Delta\eta 3 \frac{sinQR - QRcosQR}{(QR)^3} \quad (3)$$

Where R_c the inner radius which consists of solvent, t_h thickness of outer part of bilayer (head group), t_t thickness of inner part of bilayer (tail group), n_{sol}, n_h and n_t the scattering length densities of solvent, outer part of bilayer and inner part of bilayer.

Langmuir monolayers at liquid-air interface

A Langmuir film balance 302MC NIMA was used to study spreading monolayers at liquid-air interface. In all cases the monolayer was spread from a liquid phenol/chloroform/petroleum ether 2/5/8 (vol/vol) solution using Hamilton® microliter syringe (1 mg/mL, spreading volume 200 μL). 40 min were allowed for solvent evaporation before monolayer compression. All experiments were performed at 20

°C ± 0.5 °C, temperature was controlled by circulating water, the compression speed was set at 30 cm^2/min.

Computational Methods and Atomistic Models.

All molecules were represented using fully atomistic models with explicit inclusion of hydrogen atoms. The CHARMM 36 force field[9,10] was utilized for all lipids. Specifically, LipAs were modeled using an extension of CHARMM 36 customized for *E. coli* and *Chlamydia*[11,12], while model parameters for DOPE and DPSE were derived from optimized CHARMM parameters for lipids.[13,14] In all systems, the TIP3 model[15] was adopted for water molecules. Calcium and chlorine counter ions, employed to maintain system neutrality, were treated using the standard CHARMM 36 force field. The initial system coordinates were constructed through a multi-step packing process using the packmol code.[16] Initially, lipids were packed based on their area per lipid, ensuring the population of both layers of the membrane (Table 2 of main text). The packing algorithm was finely tuned to randomly insert lipids within a confined volume region, with careful attention to exposing hydrophilic lipid heads at the interface with water. The second step involved the addition of water molecules to the system. Finally, calcium and chlorine ions were randomly inserted in the vicinity of charged atoms belonging to lipids to ensure system neutrality. In Figure S5, a schematic view of the mix system composition is shown. All simulations were performed using the GROMACS package.[17] Non-bonded interactions were truncated beyond a cutoff of 1.2 Å, while Particle Mesh Ewald was employed for treating electrostatic interactions.[18] The system temperature was regulated using the velocity-rescale algorithm[19] with a time constant of 0.1 ps. A time-step of 2 fs was utilized for all simulation runs. Pressure control was achieved using the Parrinello-Rahman algorithm[20] with a semi-isotropic coupling scheme applied in the *xy*-plane (the lipid membrane plane) with a target pressure of 1.0 atm, and in the z-direction with a target pressure of 1.0 atm. A time constant of 5 ps was applied for both coupling directions. The LINCS constraint algorithm[21] was employed to constrain all bonds involving hydrogen atoms. An equilibration scheme comprising five steps was applied to all starting configurations. The first step involved a 1000-step energy minimization utilizing the steepest descent algorithm, with position restraints applied to all lipids. This restraint aimed to prevent water molecules from infiltrating the hydrophobic core of the membrane. The second step entailed a brief NVT simulation with only dihedral restraints applied to the alkyl chains of lipids. This step consisted of a 50 ps simulation at 293 K. In the third step, an additional 100 ps NVT run was conducted, during which dihedral restraints were removed. The fourth step comprised a short 1000 ps NVT run at 293 K. Finally, the fifth step involved a short equilibration in the NPT ensemble with semi-isotropic pressure coupling, utilizing the same setup for production runs of 2 μs in NPT ensemble.

Area Compressibility Calculation

To calculate the work needed to compress or expand a membrane (referred to as the Helmholtz free energy change) we used the expressions below. The work required has a quadratic dependence on the membrane area A, where A_0 represents the total area of the membrane, A denotes the size of a defect, and E_s is the isothermal area compression modulus:[22]

$$\Delta W(A) = \frac{1}{2} E_s \left(\frac{A}{A_0}\right)^2 A_0 \qquad (4)$$

From this relationship, the isothermal area compressibility can be derived as:

$$\chi_T^A = \frac{-1}{A_0}\left(\frac{\partial A}{\partial \Pi}\right)_T \equiv \frac{1}{E_s} \qquad (5)$$

Through Molecular Dynamics (MD) simulations, it is possible to directly compute the isothermal area compressibility, providing insights into membrane rigidity. Especially, membrane area fluctuations can be straightforwardly obtained from simulations and used to compute thought the expression:[23-25]

$$\chi_T^A = \frac{1}{K_B T}\left(\frac{\langle A^2 \rangle - \langle A \rangle^2}{\langle A \rangle}\right)_{N,P,T} \qquad (6)$$

In Figure S6 is shown the time-dependent behavior of the area per lipid of each lipid A built with various lipid compositions and analyzed at different temperatures.

Finally, we calculated lateral self-diffusion coefficient D_L from MD simulations as follows:[26]

$$D_L = \lim_{t \to \infty} \frac{\langle |\Delta \vec{R}_{xy}(t)|^2 \rangle}{4t} \qquad (7)$$

Where $\langle |\Delta \vec{R}_{xy}(t)|^2 \rangle$ is the mean squared displacement (MSD) computed in the *xy* membrane plane.

Supplementary Tables

Table S1. ^1H and ^{13}C (*italic*) of the chemical shift values of the O-antigen fraction obtained upon mild acid hydrolysis (**A**-**E**) of the LPS from both *Pseudomonas* grown at 0.4 °C and 20 °C.

	**1**	**2**	**3**	**4**	**5**	**6**
**A**	4.95	3.88	3.80	3.59	3.97	3.70
4-α-D-GalNAc	*94.0*	*53.8*	*69.4*	*77.2*	*71.1*	*59.9*
		C=O *174.9*<br>CH_3 2.06/*22.4*				
**B**	4.91	3.67	3.37	3.37	3.37	1.27
4-β-L-QuiNAc	*101.7*	*55.8*	*73.6*	*82.0*	*70.7*	*17.1*
		C=O *174.6*<br>CH_3 1.97/*22.1*				
**C**	4.91	3.60	4.13	3.14	4.09	1.14
t-α-D-Qui3NAc	*96.0*	*70.2*	*53.8*	*72.9*	*68.3*	*16.5*
			C=O *175.2*<br>CH_3 1.99/*22.1*			
**D**	4.87	4.14	3.72	3.27	3.90	1.16
2,3-α-L-Rha	*99.8*	*75.1*	*76.7*	*70.6*	*69.3*	*16.7*
**E**	4.77	4.09	3.54	3.32	3.32	1.26
3-β-L-Rha	*100.5*	*67.3*	*77.6*	*70.4*	*72.1*	*16.9*

Table S2. The main ion peaks observed in the MALDI-TOF MS spectrum reported in Figure 2, the predicted masses, and the proposed interpretation of the lipid A species. The observed masses reported in the table are compared to the calculated monoisotopic mass (predicted mass, Da) of each ion based on the proposed structures.

**Predicted mass (Da)**	**Observed ion peaks (*m/z*)**	**Acyl substitution**	**Proposed composition**
1792.9	1792.2	Hexa-acyl	$HexN^{2}$, P^{4}, $[12:0(OH)]^{4}$, $[10:0(3\text{-}OH)]^{2}$
1756.0	1755.1	Hexa-acyl	$HexN^{2}$, P^{3}, $[12:0(OH)]^{4}$, $[10:0(3\text{-}OH)]^{2}$, EtN
1740.0	1739.2	Hexa-acyl	$HexN^{2}$, P^{3}, $[12:0(OH)]^{3}$, $[10:0(3\text{-}OH)]^{2}$, 12:0, EtN
1632.9	1632.1	Hexa-acyl	$HexN^{2}$, P^{2}, $[12:0(OH)]^{4}$, $[10:0(3\text{-}OH)]^{2}$
1617.0	1616.1	Hexa-acyl	$HexN^{2}$, P^{2}, $[12:0(OH)]^{3}$, $[10:0(3\text{-}OH)]^{2}$, 12:0
1601.0	1600.1	Hexa-acyl	$HexN^{2}$, P^{2}, $[12:0(OH)]^{2}$, $[10:0(3\text{-}OH)]^{2}$, $(12:0)^{2}$
1553.0	1552.1	Hexa-acyl	$HexN^{2}$, P, $[12:0(OH)]^{4}$, $[10:0(3\text{-}OH)]^{2}$
1537.0	1536.1	Hexa-acyl	$HexN^{2}$, P, $[12:0(OH)]^{3}$, $[10:0(3\text{-}OH)]^{2}$, 12:0
1521.0	1520.1	Hexa-acyl	$HexN^{2}$, P, $[12:0(OH)]^{2}$, $[10:0(3\text{-}OH)]^{2}$, $(12:0)^{2}$
1462.9	1462.0	Penta-acyl	$HexN^{2}$, P^{2}, $[12:0(OH)]^{4}$, $[10:0(3\text{-}OH)]$
1446.9	1446.0	Penta-acyl	$HexN^{2}$, P^{2}, $[12:0(OH)]^{3}$, $[10:0(3\text{-}OH)]$, 12:0
1382.9	1382.0	Penta-acyl	$HexN^{2}$, P, $[12:0(OH)]^{4}$, $[10:0(3\text{-}OH)]$
1350.9	1350.0	Penta-acyl	$HexN^{2}$, P, $[12:0(OH)]^{2}$, $[10:0(3\text{-}OH)]$, $(12:0)^{2}$

Table S3. Fitting parameters from SAXS data fitted with bilayered vesicle model from Sasfit. Where Rc is the radius of vesicle core which consists of solvent, t_h is thickness of outer part of bilayer (in contact with solvent, head group), t_t is thickness of inner part of bilayer (tail group), η_{sol} is the scattering length density of the solvent, η_h is the scattering length density of the outer part of bilayer, and η_t is the scattering length density of the inner part of bilayer. Rc has been estimated 80 nm from TEM images and it doesn't affect the scattering intensity in the analyzed q-range. η_{sol}, η_h, and η_t values have been used as reported in literature. According to the employed fitting, the total bilayer thickness corresponds to the sum of the layer of the hydrophobic tails and the two (internal and external) layers of the hydrophilic heads in contact with the water.[27]

	Rc (nm)	t_h (nm)	t_t (nm)	η_{sol}($x10^{-4}nm^{-2}$)	η_h($x10^{-4}nm^{-2}$)	η_t($x10^{-4}nm^{-2}$)
**$LPS_{0.4C}$**	80	2.3 ± 0.1	2.8 ± 0.1	9.4	14	8.8
**LPS_{20C}**	80	2.2 ± 0.1	2.8 ± 0.1	9.4	14	8.8

Supplmentary Figures

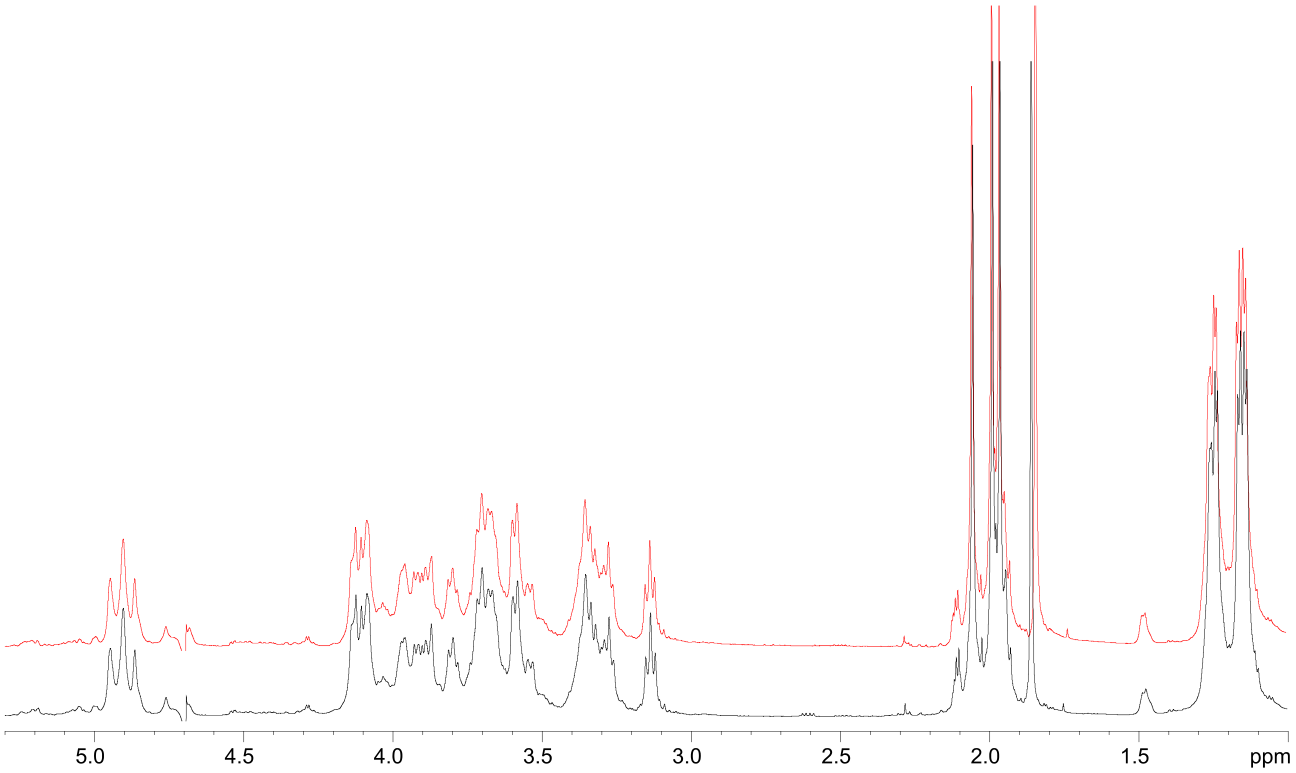

Figure S1. (600 MHz, 298 K, D_2O, Table S1). ^{1}H NMR spectra of the O-antigen fraction obtained upon mild acid hydrolysis of the *Pseudomonas* sp. EW#7 $\mathbf{LPS_{0.4C}}$ (red) and $\mathbf{LPS_{20C}}$ (black)

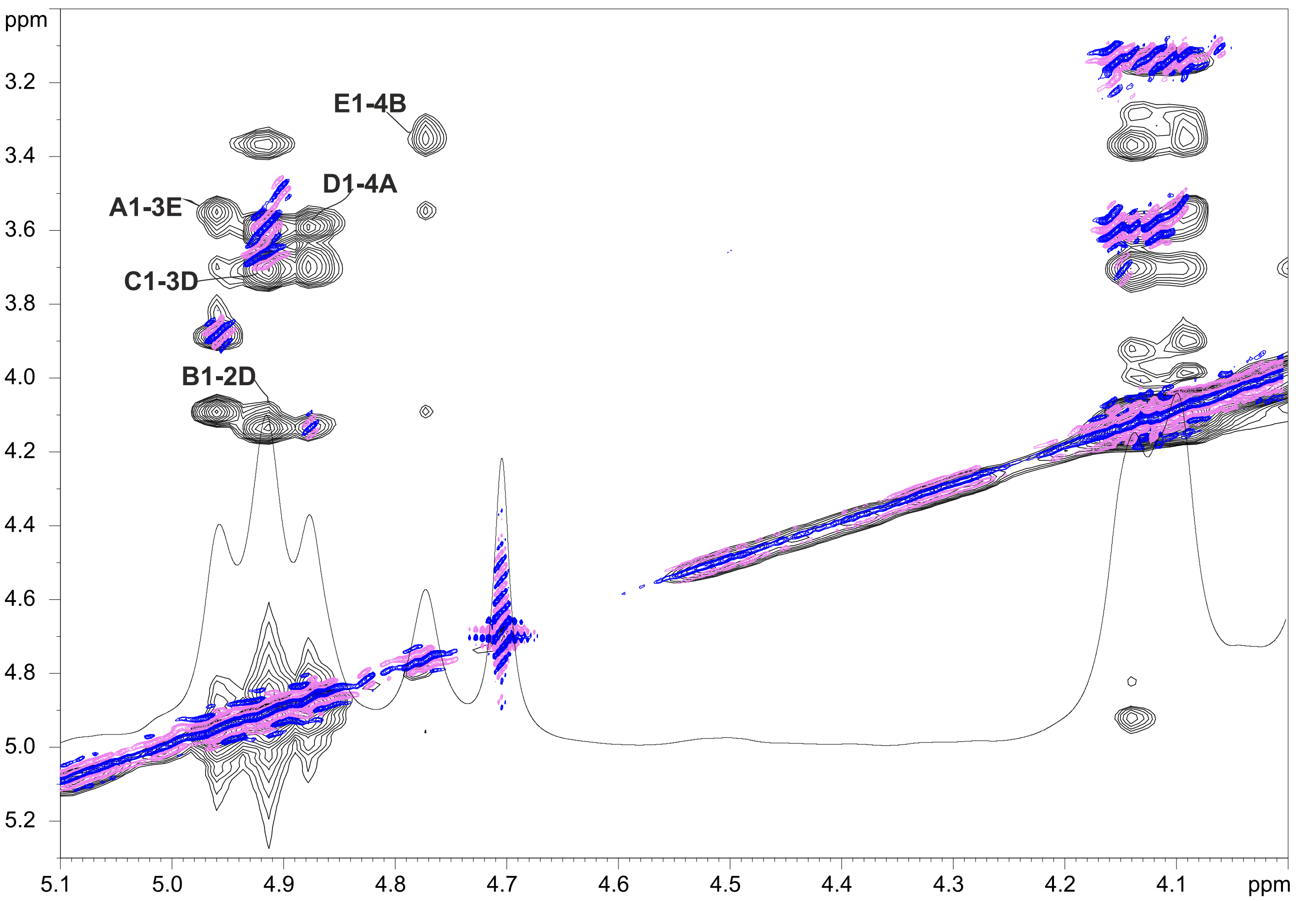

Figure S2. (600 MHz, 298 K, D_2O, Table S1). ^1H, NOESY (black) and COSY (pink and blue) spectra of the O-antigen from *Pseudomonas* sp. EW#7 $\mathbf{LPS_{0.4C}}$

Figure S3. Negative-ion MALDI MS/MS spectra of precursor ions at *m/z* 1632.1 (top) and *m/z* 1616.1 (bottom), chosen as representatives of the predominant species detected in MALDI-TOF MS spectra of lipid A from $\mathbf{LPS_{20C}}$ and $\mathbf{LPS_{0.4C}}$ respectively. Both matched with *bis*-phosphorylated hexa-acylated lipid A species, but they differ for secondary acylation. The assignment of main fragments is reported. In each inset the proposed structure has been depicted. Peaks originated from the loss of $C_{10}H_{20}O$ (156 mass units), which is caused by a rearrangement occurring on *N*-linked 3-OH acyl chains having the hydroxyl group free, have been also indicated. The concomitant presence of these peaks, as well as those derived from glycosidic linkage cleavage (Y_1) and from cross-ring fragmentations ($^{1,4}A_2$) guided the positioning of both the secondary acyl chains and the phosphate groups.

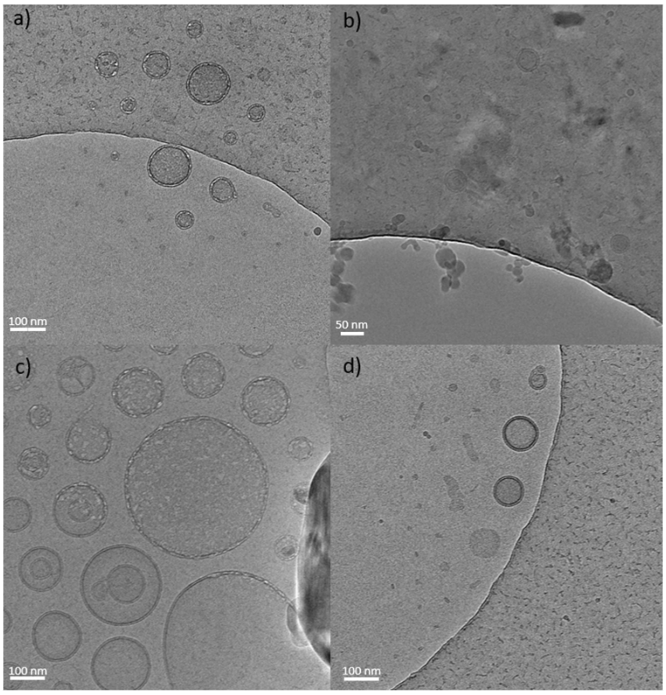

Figure S4. Cryo-EM micrographs of 1 mg/mL LPS water solution showing the presence of vesicles and micelle-like structures.

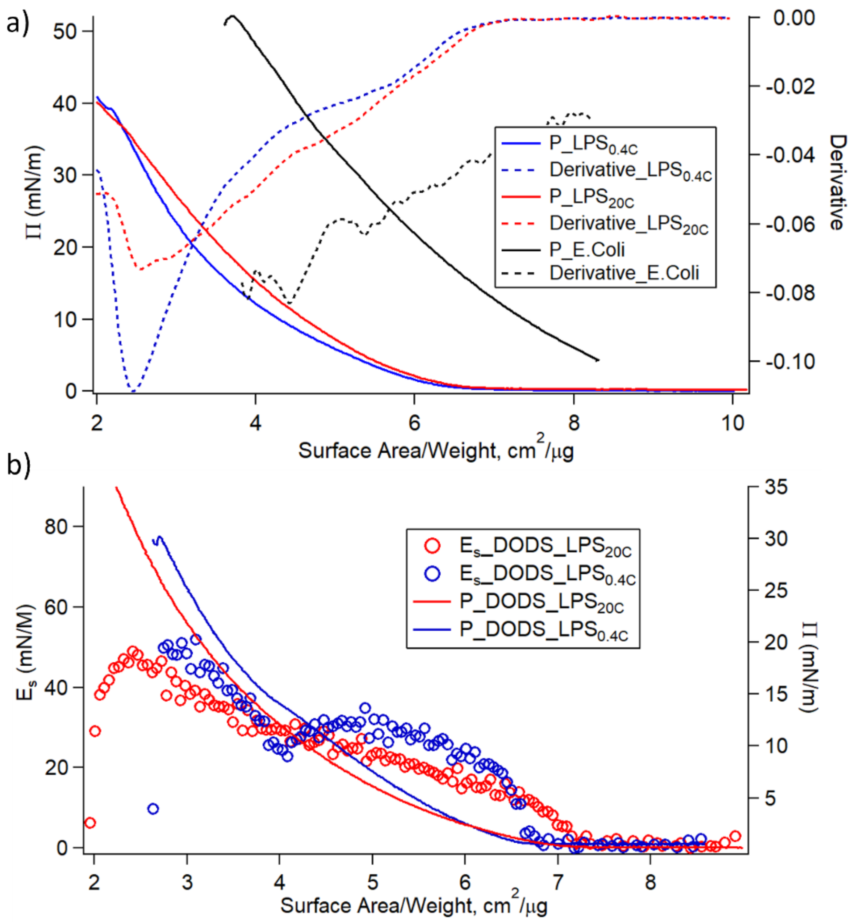

Figure S5. (**a**) Surface pressure-area isotherms (solid lines) and curves derivatives (dashed lines) of **LPS_{20C}** mixture (red), **$LPS_{0.4C}$** (blue), and LPS from *E. coli* (black); (**b**) Compression modulus (E_s) as a function of area per unit weight (A) for DOPE-DSPE-**LPS_{20C}** mixture (red) and DOPE-DSPE-**$LPS_{0.4C}$** (blue).

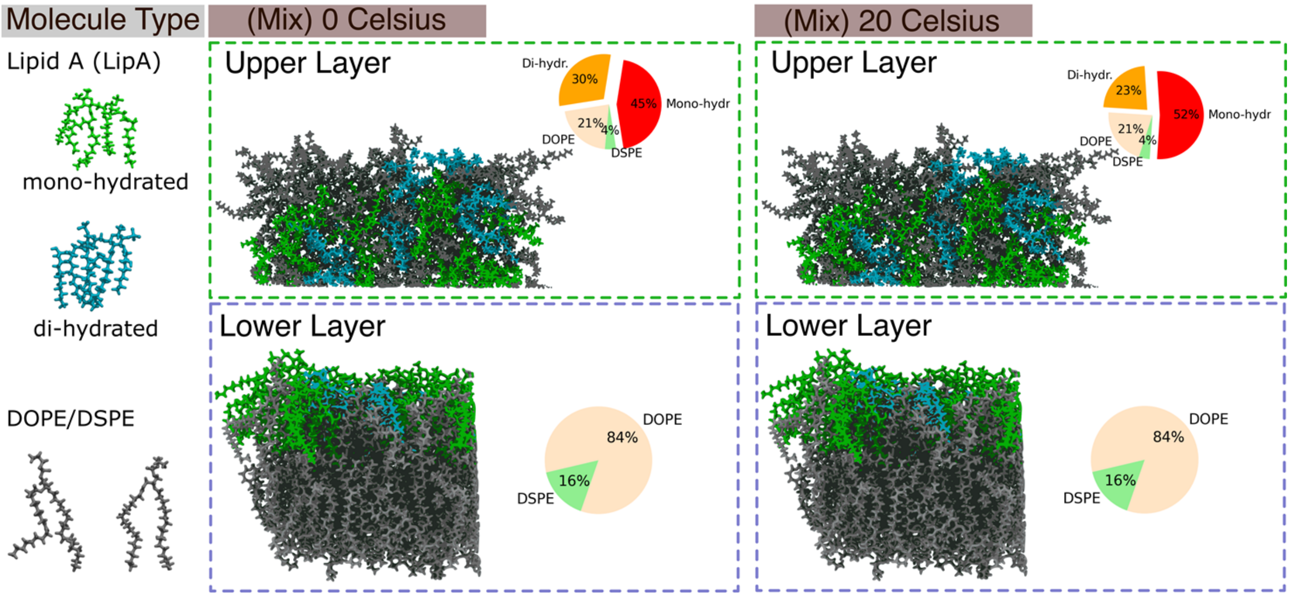

Figure S6. Schematic representation of the upper- and lower-layers compositions for the mix systems simulated at 0 °C and 20 °C.

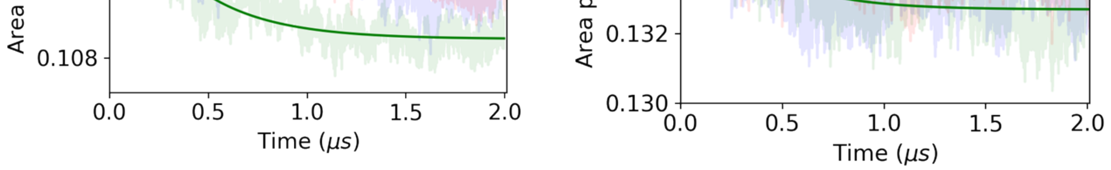

Figure S7. Time behavior of area/lipid A_0 calculated for systems at: (A) 0°C and (B) 20 °C.

Supplementary References

1. O. Westphal, O. Lüderitz, F. Bister, B. Naturforsch, *J. Chem. Sci.*, 1952, **7**, 148–155.
2. M. D. Pither, A. Silipo, A. Molinaro, F. Di Lorenzo, *Methods Mol. Biol.*, 2023, **2613**, 153-179.
3. P. Garcia-Vello, I. Speciale, F. Di Lorenzo, A. Molinaro, C. De Castro, *Methods Mol. Biol.*, 2022, **2548**, 181-209H.
4. E. T. Rietschel, *Eur. J. Biochem.*, 1976, **64**, 423–428.
5. E. G. Bligh, W. J. Dyer, J. Can., *Biochem. Physiol.*, 1959, **37**, 911–917.

6. F. Di Lorenzo, L. Paparo, L. Pisapia, F. Oglio, M.D. Pither, R. Cirella, R. Nocerino, L. Carucci, A. Silipo, F. de Filippis, D. Ercolini, A. Molinaro, R. Berni Canani, *Front. Mol. Biosci.*, 2023, **10**, 1266293.
7. E. Andretta, S. De Chiara, C. Pagliuca, R. Cirella, E. Scaglione, M. Di Rosario, M.S. Kokoulin, O.I. Nedashkovskaya, A. Silipo, P. Salvatore, A. Molinaro, F. Di Lorenzo, *Glycoconj. J.*, 2024, **41**, 119–131.
8. D. Vollhardt, V. B. Fainerman, *Adv. Colloid Interface Sci.*, 2006, **127**, 83-97.
9. J. Huang, A. D. Jr. MacKerell, *J. Comput. Chem.*, 2013, **34**, 2135-45.
10. T. J. Piggot, Á. Piñeiro, S. Khalid, *J. Chem. Theory Comput.*, 2012, **13**, 4593-609.
11. K. R. Pandit & J. B. Klauda, *Biochim. Biophys. Acta (BBA) – Biomembr.*, 2012, **1818**, 1205-1210.
12. J.B. Lim, & J.B. Klauda, *Biochim. et Biophys. Acta (BBA) – Biomembr.*, 2011, **1808**, 323-331.
13. J. B. Klauda, R. M. Venable, J. A. Freites, J. W. O'Connor, D. J. Tobias, C. Mondragon-Ramirez, I. Vorobyov, A. D. MacKerell, Jr. and R. W. Pastor, *J. Phys. Chem. B.*, 2010, **114**, 7830-7843.
14. J. B. Klauda, V. Monje, T. Kim, and W. Im, *J. Phys. Chem. B.*, 2012, 116, 9424-9431.
15. W. L. Jorgensen, J. Chandrasekhar, J. D. Madura, R. W. Impey, M. L. Klein, *J. Chem. Phys.*, 1983, **79**, 926–935.
16. L. Martínez, R. Andrade, E. G. Birgin, J. M. Martínez. Packmol, *J. Computat. Chem.*, 2009, **30**, 2157-2164.
17. D. van der Spoel, E. Lindahl, B. Hess, G. Groenhof, A.E. Mark, and H. J. C. Berendsen, *J. Comp. Chem.*, 2005, **26**, 1701–1718.
18. T. Darden, D. York, and L. Pedersen, *J. Chem. Phys.*, 1993, **98**, 10089–10092.
19. G. Bussi, D. Donadio, and M. Parrinello, *J. Chem. Phys.*, 2007, **126**, 014101.
20. M. Parrinello and A. Rahman, *J. Appl. Phys.*, 1981, **52**, 7182–7190.
21. B. Hess, H. Bekker, H. J. C. Berendsen, and J. G. E. M. Fraaije, *J. Comp. Chem.*, 1997, **18,** 1463–1472.
22. T. Heimburg, *Biochim. Biophys. Acta*, 1998, **1415**, 147-162.
23. A. Blicher, K. Wodzinska, M. Fidorra, M. Winterhalter, T. Heimburg, *Biophys. J.*, 2009, **96**, 4581-91.
24. M. M. Terzi, M. Deserno, J. F. Nagle, *Soft Matter.*, 2019, **15**, 9085–9092.
25. Y. Andoh, S. Okazaki, R. Ueoka, *Biochim. Biophys. Acta*, 2013, **1828**, 1259-70.
26. D. Frenkel and B. Smit, Understanding Molecular Simulation: From Algorithms to Applications, Academic Press; 2nd edition; 2001.
27. E. F. Semeraro, L. Marx, J. Mandl, M. P. K. Frewein, H.L. Scott, S. Prévost, H. Bergler, K. Lohner, G. Pabst, *J. Appl. Crystallogr.*, 2021, **54**(Pt 2), 473-485.
